# Effects of omega-3 fatty acids on patients undergoing surgery for gastrointestinal malignancy: a systematic review and meta-analysis

**DOI:** 10.1186/s12885-017-3248-y

**Published:** 2017-04-14

**Authors:** Jing Yu, Lian Liu, Yue Zhang, Jia Wei, Fan Yang

**Affiliations:** Department of Oncology, Beijing Friendship Hospital, Capital Medical University, No. 95 Yong An Road, Xicheng District, Beijing, 100050 China

**Keywords:** Omega-3 fatty acids, Immune function, Gastrointestinal malignancy, Postoperative complications

## Abstract

**Background:**

Surgical resection remains the primary treatment for gastrointestinal (GI) malignancy including early-stage cancer. Omega-3 polyunsaturated fatty acids (n-3 PUFAs) have been reported to have beneficial clinical and immune-modulating effects in the prognosis of GI cancer patients undergoing surgery.

**Methods:**

We searched PubMed, Embase, EBSCO-Medline, Cochrane Central Register of Controlled Trials (CENTRAL), CNKI and Wanfang to identify primary research reporting the effects of n-3 PUFAs compared with isocaloric nutrition on GI cancer patients who underwent surgery up to the end of June 30, 2016. Two authors independently reviewed and selected eligible randomized controlled trials (RCTs).

**Results:**

A total of 9 RCTs (623 participants) were included. The n-3 PUFAs regime resulted in lower levels of C-reactive protein (CRP) (*P* < 0.05), interleukin-6 (IL-6) (*P* < 0.01), and higher levels of albumin (ALB), CD3^+^ T cells, CD4^+^ T cells and CD4^+^/CD8^+^ ratio (*P* < 0.05) compared with the isocaloric nutrition regime. However, there was no significant difference in the level of tumor necrosis factor-α (TNF-α) between the n-3 PUFAs regime and the isocaloric nutrition regime (*P* = 0.17). And the level of CD8 ^+^ T cells decreased compared with the isocaloric nutrition regime (*P* < 0.0001).

**Conclusions:**

Our meta-analysis revealed that n-3 PUFAs are effective in improving the nutritional status and immune function of GI cancer patients undergoing surgery as they effectively enhance immunity and attenuate the inflammatory response.

## Background

GI cancers are the most common group of malignancies and many types of GI cancer are ranked as the leading cause of cancer death worldwide [1, 2]. Surgery is the primary treatment for patients with early-stage GI cancer. However, patients undergoing selective GI cancer surgery will face the risk of developing various postoperative complications due to negative impact factors, such as malnutrition, tumor-induced immune suppression, surgical stress, and inflammation.

Postoperative complications affect the clinic outcome of patients, resulting in prolonged hospital-stay and increased costs. Of these complications, malnutrition is the most important factor influencing clinical prognosis [3, 4].

Current studies indicate that nutritional support can reduce the incidence of adverse events after major GI surgery. Omega-3 polyunsaturated fatty acids (n-3 PUFAs) modulate the level of inflammation and reduce oxidative stress and complications [5–8]. The evidence from these studies indicates that n-3 PUFAs have an anti-inflammatory effect, which promotes wound healing, and enhances the adaptive immune response [9, 10]. However, interpretation of these studies is problematic due to methodological limitations and small sample sizes. Moreover, the results of several recent RCTs are controversial. Thus, the purpose of this systematic review is to evaluate the potential role of n-3 PUFAs in the outcome of GI cancer patients after surgery.

## Methods

### Research design

We searched PubMed (January 1, 1976, through April 30, 2016), EMBASE (January 1, 1985, through April 30, 2016), the Cochrane Library (January 1, 1987, through April 30, 2016), CNKI (January 1, 1986, through April 30, 2016), Wanfang (January 1, 1985, through April 30, 2016) and VIP databases (January 1, 1985, through April 30, 2016) using common keywords related to n-3 PUFAs and GI cancer. The following key words were included: n-3 PUFAs, eicosapentaenoic acid or EPA, docosahexaenoic acid or DHA, gastrointestinal malignancy or cancer surgery. We reviewed the bibliographies of relevant articles for additional publications.

### Selection criteria

We included trials that met the following four criteria: (1) the trial enrolled adult patients (male or female aged at least 18 years) undergoing surgery for GI malignancy; (2) the trial design was randomized, double blind, and placebo-controlled; (3) the trial compared n-3 PUFAs support with isocaloric nutrition; (4) the trial reported outcome measures such as CD3^+^ T cells, CD4^+^ T cells, CD8^+^ T cells, CD4^+^/CD8^+^ T cells, ALB, IL-6, TNF-α, and CRP; (5) the study did not include obese patients and there was no difference in body mass index (BMI) between the groups.

### Data extraction

Two co-first authors reviewed all the articles independently and discussed the articles until a consensus was reached. Data obtained from the studies included the first author, year of publication, patient source (region), tumor types, and type of study. All data were extracted independently by two investigators. As all the studies were RCTs, we summarized the basic parameters and then assessed the quality of the included studies.

### Quality evaluation

We assessed the methodological quality of the included studies using the scale of Risk of bias summary and Risk of bias graph, which is the most widely used assessment tool in meta analyses. The scale measures the following characteristics in RCTs: 1. random sequence generation (selection bias), 2. allocation concealment (selection bias), 3. blinding method used for participants and study personnel (performance bias), 4. blinding method used for outcome assessment (detection bias), 5. incomplete outcome data (attrition bias), 6. selective reporting (reporting bias), 7. other biases. The risk of each included study was rated as “high bias risk”, “unclear bias risk” or “low bias risk” according to the information extracted. The graphical results of methodological quality are shown in Fig. 2.

### Statistical analysis

The levels of CRP, IL-6 and TNF-α, ALB, CD3^+^T cells, CD4^+^T cells, CD8^+^T cells, and CD4^+^/CD8^+^T cells were calculated using the Review Manager 5.0.24 statistical software (Cochrane Collaboration Software). Publication bias was evaluated according to a funnel plot and Begg’s and Egger’s tests using the Review Manager 5.0.24 package. Heterogeneity was considered statistically significant when *P* < 0.05.

## Results

### Characteristics of the included studies and risk of bias

**Fig. 1:**
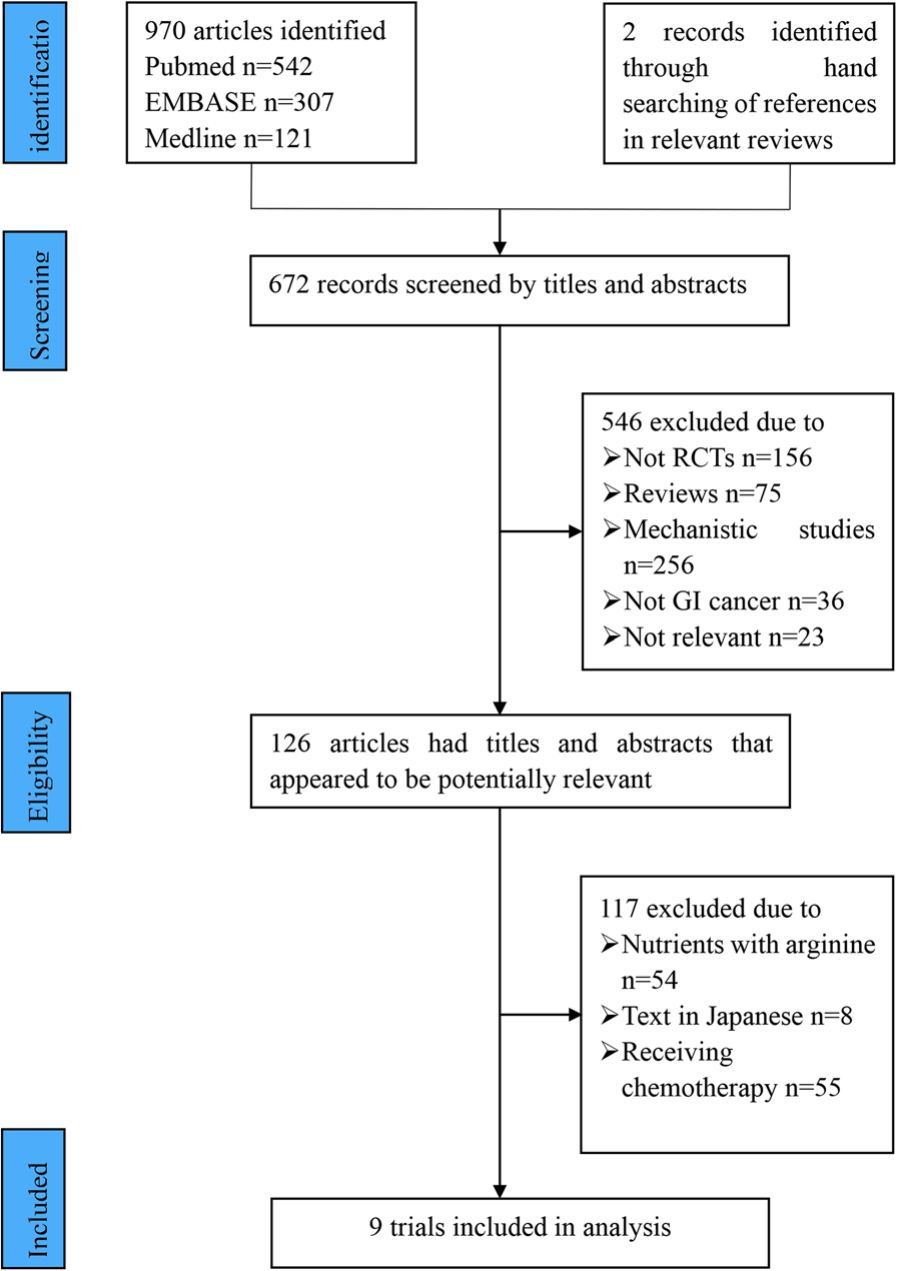
Flowchart of computerized search and the eligible studies included in this systematic review and meta-analysis

The electronic literature search yielded 672 potential studies for inclusion. And finally, 126 articles had titles and abstracts that appeared to be potentially relevant. Of these studies, 54 studies were excluded because the patients received arginine. 8 studies were reported neither in Chinese nor in English and were thus excluded; 55 studies with full texts were further excluded as the patients received chemotherapy. All procedures were performed by two investigators independently. In total, 9 eligible studies were included in this meta-analysis. The flow chart of retrieval and selection of the studies is shown in Fig. 1.

Table 1 summarizes the basic characteristics of the included studies. Of the 8 studies included, 5 trials reported the association between fish oil consumption and the level of CRP [11–15], 5 trials described the correlation between PUFAs and the level of IL-6 [11, 12, 14, 16, 17], 4 trials investigated the association between n-3 PUFAs and the level of TNF-α [11, 12, 14, 16], 4 trials investigated the association between n-3 PUFAs and the level of ALB [12–14, 17], 7 trials investigated the association between n-3 PUFAs and the level of inflammation [11–17], and 7 trials described the correlation between n-3 PUFAs and immune functions [12–16, 18, 19]. Nutritional status was classified by the Nutritional Risk Index (NRI). If the NRI was >100, the patient was not considered malnourished, 97.5–100 indicated mild malnutrition, 83.5–97.5 indicated moderate malnutrition, and <83.5 indicated severe malnutrition. However, there was no significant difference between the groups in terms of mean weight and BMI in the included studies. Of the included studies, 6 studies were from China [11, 13–16, 18], one study was from Brazil [12], one study was from UK [19], and one study was from Ireland [17].

**Table 1:** Characteristics of included randomized trials

Trial(year)	Time	Country	Intervention	n-3 PUFA,n	Contro l, n	Age	BMI	Cancer	Studu design	Parameters
Ma et al. [12]	2015	China	0.8–1.5 g/kg/d LCT,MCT, n-3PUFA	44	41	61.55 ± 9.78	23.45 ± 3.44 44	stomach, colon	Randomized	IL-6, CRP, TNF-α
Ziran et al. [13]	2014	Brazil	104–125 kJ/kg/d EPA + DHA	26	20	36–74	17.8–29.7	stomach	Randomized	CD3, CD4, CD8, CD4/CD8, albumin, L-6, CRP, TNF-α
Liu et al. [14]	2009	China	83.68 kJ/kg/d n-3PUFA	22	20	64.02	Not Given	stomach, colon	Randomized	CD3, CD4, CD8, CD4/CD8, albumin, CRP
Hu et al. [15]	2015	China	104–125 kJ/kg/d n-3PUFA	44	44	23–78	Not Given	stomach, colon	Randomized	CD3, CD4, CD8, CD4/CD8, IL-6, CRP, TNF-α
Zheng et al. [16]	2011	China	25 kcal//kg/d n-3PUFA	20	20	56.19 ± 11.80	Not Given	colon, rectum	Randomized	CD3, CD4, CD4/CD8, albumin, CRP
ZHU et al. [17]	2012	China	1.2 g/kg/d n-3PUFA	29	28	70.8 ± 6.4	18.5–25.0	colon	Randomized	CD4, CD8, CD4/CD8, IL-6, TNF-α
Aoife et al. [18]	2009	Ireland	2.2 g/d EPA	28	25	62	24.6	esophagus	Randomized	albumin, IL-6
Huang et al. [19]	2014	China	25 kcal//kg/d n-3PUFA	40	40	60.88 ± 7.54	Not Given	esophagus	Randomized	CD3, CD4, CD8, CD4/CD8
Sultan et al. [20]	2012	UK	EPA 0·51 g/100 ml + DHA 0·22 g /100 ml	66	66	67 (42–79)	Not Given	esophagus, stomach	Randomized	CD3, CD4, CD8, CD4/CD8

*LCT* long-chain triglyceride, *MCT* medium-chain triglyceride

**Fig. 2:**
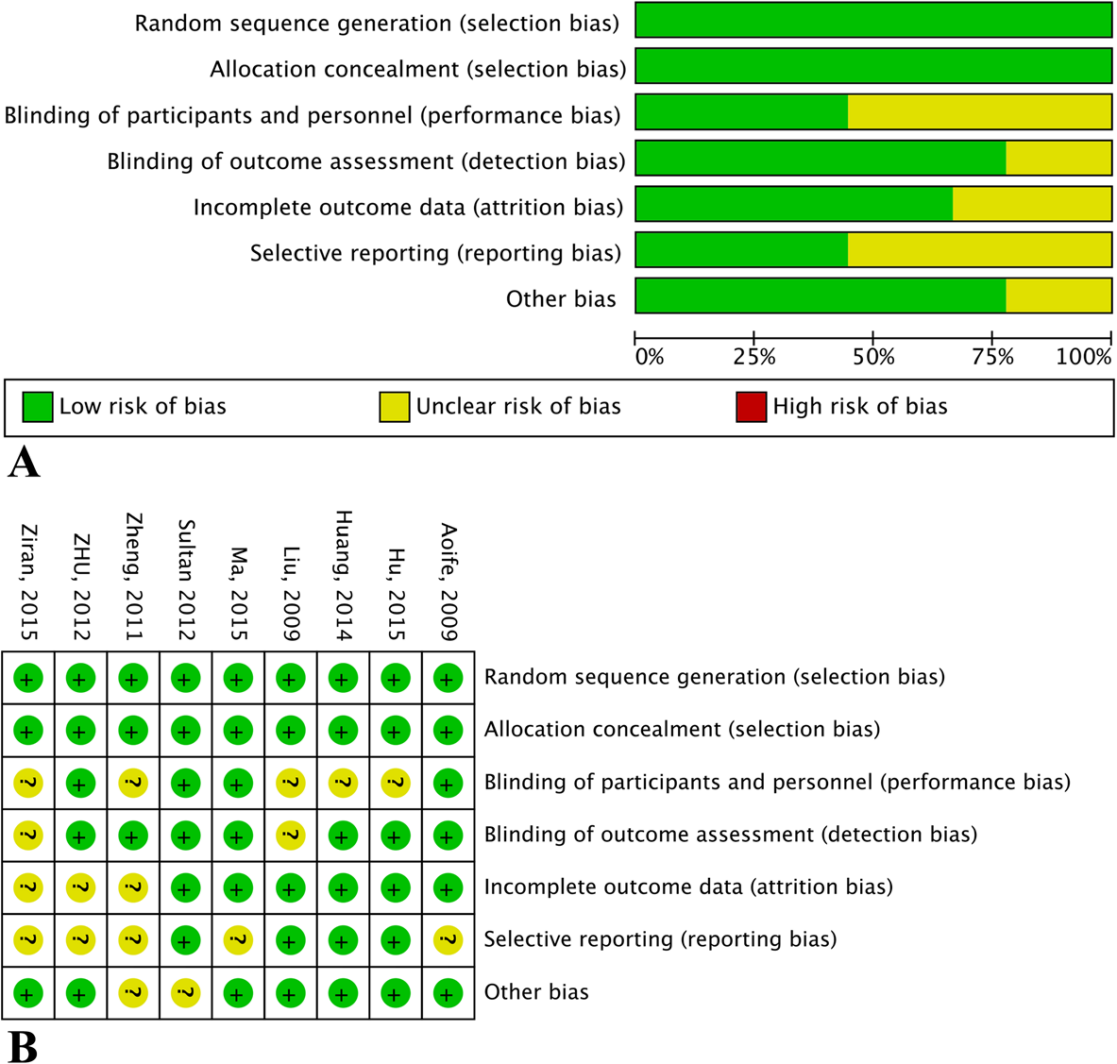
Assessment of risk of bias based on the evaluation domains listed in the Cochrane Collaboration Risk of Bias Tool: risk of bias graph (**a**), risk of bias summary (**b**)

All 9 studies were double-blind and allocation concealment was adequate in all studies. The risk of bias items for each included study are presented in Fig. 2.

### Level of inflammation

**Fig. 3:**
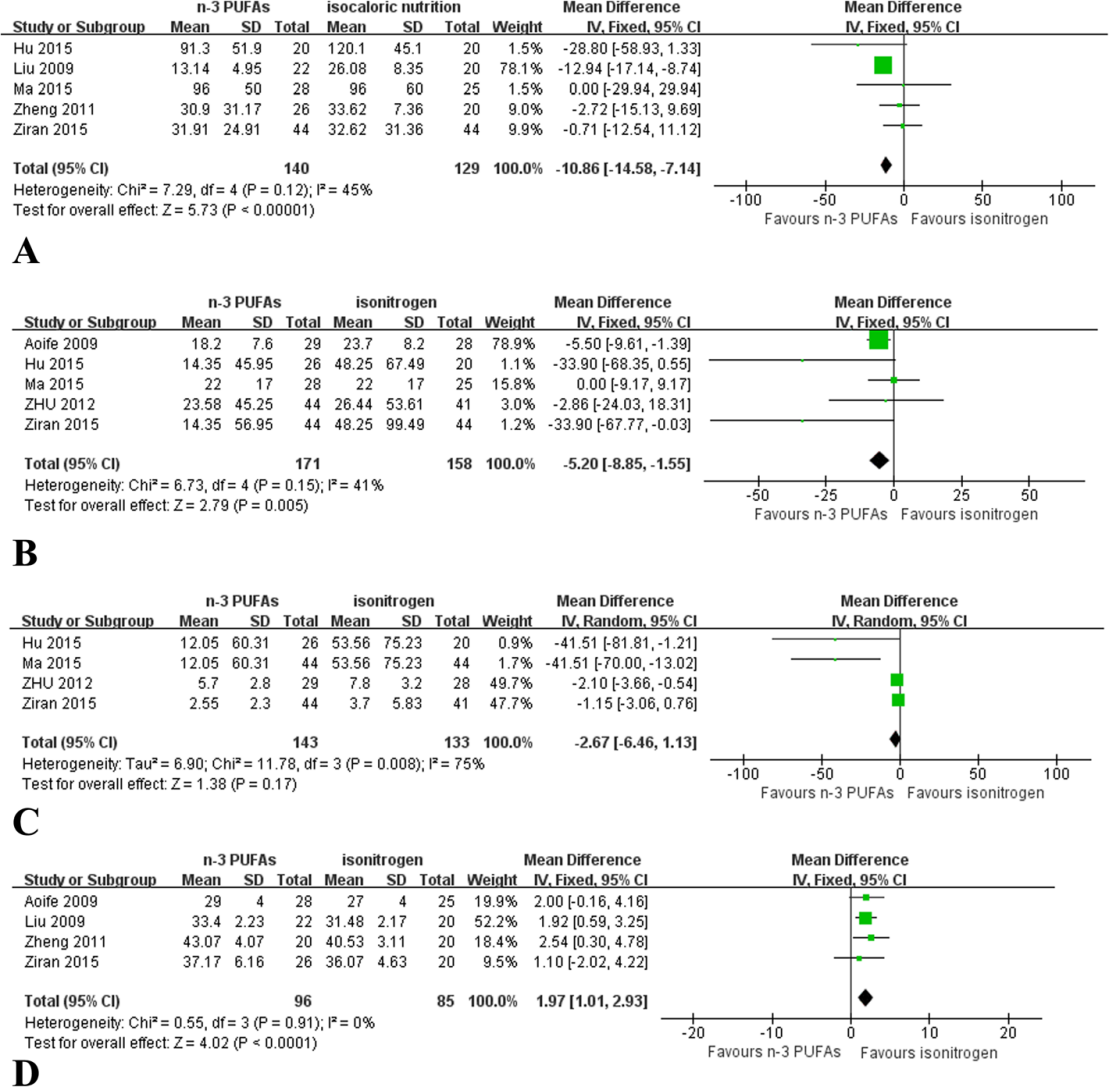
Meta-analysis of inflammation level. **a** Change in CRP between n-3 PUFAs and isocaloric nutrition: random-effects model. **b** Change in IL-6 between n-3 PUFAs and isocaloric nutrition: random-effects model. **c** Change in TNF-a between n-3 PUFAs and isocaloric nutrition: random-effects model. **d** Change in ALB between n-3 PUFAs and isocaloric nutrition: random-effects model

CRP: We identified 5 eligible trials, which included 269 patients, and investigated peripheral blood CRP levels following postoperative n-3 PUFAs supplementation versus isocaloric nutrition. The homogeneous test detected no statistical heterogeneity (*P* = 0.12), therefore, we adopted a fixed-effects model to perform the analysis. The meta-analysis revealed that n-3 PUFAs effectively decreased the level of CRP (*P* < 0.05) (Fig. 3).

IL-6: We identified 5 eligible trials, which included 329 patients, and investigated IL-6 levels following postoperative n-3 PUFAs supplementation versus isocaloric nutrition. The homogeneous test detected no statistical heterogeneity (*P* = 0.15), therefore, we adopted a fixed-effects model to perform the analysis. The meta-analysis revealed that n-3 PUFAs effectively decreased the level of IL-6 (*P* = 0.005) (Fig. 3).

TNF-α: We identified 4 eligible trials, which included 276 patients, and investigated TNF-α levels following postoperative n-3 PUFAs supplementation versus isocaloric nutrition. The homogeneous test detected substantial statistical heterogeneity (*P* = 0.008), therefore, we adopted a random-effects model to perform the analysis. The meta-analysis revealed that TNF-α levels decreased following both n-3 PUFAs supplementation and isocaloric nutrition; however, there was no significant difference in TNF-α level between the two treatment groups (*P* = 0.17) (Fig. 3).

ALB: We identified 4 eligible trials, which included 181 patients, and investigated ALB levels following postoperative n-3 PUFAs supplementation versus isocaloric nutrition. The homogeneous test detected no statistical heterogeneity (*P* = 0.91), therefore, we adopted a fixed-effects model to perform the analysis. The meta-analysis revealed that n-3 PUFAs effectively increased the level of ALB (*P* < 0.01) (Fig. 3).

### Immune status

**Fig. 4:**
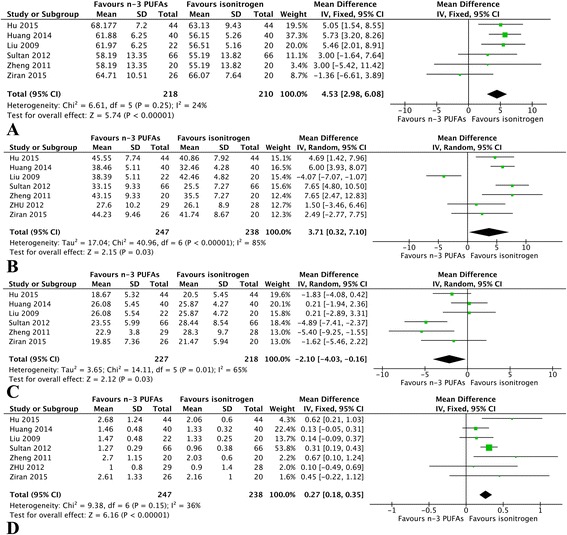
Meta-analysis of immune indices. **a** Pooled results of CD3^+^Tcells between n-3 PUFAs and isocaloric nutrition: fixed-effects model. **b** Change in CD4^+^T cells between n-3 PUFAs and isocaloric nutrition: random-effects model. **c** Change in CD8^+^T cells between n-3 PUFAs and isocaloric nutrition: random-effects model. **d** Change in CD4^+^/CD8^+^T cells between n-3 PUFAs and isocaloric nutrition: fixed-effects model

CD3^+^T cells: We identified 6 eligible trials, which included 428 patients, and investigated CD3^+^T cell levels following postoperative n-3 PUFAs supplementation versus isocaloric nutrition. The homogeneous test detected no statistical heterogeneity (*P* = 0.25), therefore, we adopted a fixed-effects model to perform the analysis. The meta-analysis revealed that n-3 PUFAs effectively increased the level of CD3^+^T cells (*P* < 0.01) (Fig. 4).

CD4^+^ T cells: We identified 7 eligible trials, which included 485 patients, and investigated CD4^+^T cell levels following postoperative n-3 PUFAs supplementation versus isocaloric nutrition. The homogeneous test detected substantial statistical heterogeneity (*P* < 0.00001), therefore, we adopted a random-effects model to perform the analysis. The meta-analysis revealed that n-3 PUFAs effectively increased the level of CD4^+^T cells (*P* = 0.03) (Fig. 4).

CD8^+^ T cells: We identified 6 eligible trials, which included 445 patients, and investigated CD8^+^T cell levels following postoperative n-3 PUFAs supplementation versus isocaloric nutrition. The homogeneous test detected substantial statistical heterogeneity (*P* = 0.01), therefore, we adopted a random-effects model to perform the analysis. The meta-analysis revealed that n-3 PUFAs effectively decreased the level of CD8^+^T cells (*P* = 0.03) (Fig. 4).

CD4^+^/CD8^+^ T cells: We identified 7 eligible trials, which included 485 patients, and investigated CD4^+^/CD8^+^ levels following postoperative n-3 PUFAs supplementation versus isocaloric nutrition. The homogeneous test detected no statistical heterogeneity (*P* = 0.15); therefore, we adopted a fixed-effects model to perform the analysis. The meta-analysis revealed that n-3 PUFAs effectively increased the level of CD4^+^/CD8^+^ T cells (*P* < 0.00001) (Fig. 4).

### Publication bias

There was no evidence of publication bias following assessment by funnel plot, Egger’s test (*P* > 0.05) and Begg’s test (*P* > 0.05).

## Discussion

The ASPEN guide recommends that for patients with large tumors undergoing surgery, a variety of immune nutrients in the nutritional formulation are conducive for improving prognosis. It is best to start nutritional support 5–7 days before surgery, and it should be continued into the postoperative period [20]. N-3 PUFAs have been reported to have a role in enhancing host immunity and attenuating the inflammatory response in GI cancer patients undergoing surgery [21]. There is evidence to suggest that n-3 PUFAs play an important role in the host immune response and inflammatory reaction in GI cancer, thus n-3 PUFAs are the best option for postoperative management compared with isocaloric nutrition [22–25].

We conducted a systematic review based on eight RCTs involving 583 patients and evaluated the impact of n-3 PUFAs on postoperative inflammation status and immune function. The results of our study showed that n-3 PUFAs significantly decreased the level of inflammation and increased immune function.

N-3 PUFAs are beneficial as a dietary supplement for cancer patients as they reduce the level of inflammatory cytokines, including IL-2, IL-6, as well as TNF-α, and promote anti-inflammatory activities. IL-6, an inflammatory cytokine, can down-regulate the stress response, and mainly originates from immune cells (e.g., T cells), endotheliocytes, and macrophages. It can effectively modulate the immune system and fight infection. Serum ALB is a negative acute phase protein and ALB concentration has important roles in the regulation of inflammation [24], while CRP is a marker of acute inflammation. Many previously published studies have revealed that n-3 PUFAs can down-regulate the levels of IL-6 and TNF-α in cancer patients postoperatively [26–30]. The trial by Turnocket al. revealed that perioperative administration of n-3 PUFAs suppressed the level of CRP in patients undergoing surgery for GI malignancy [31]. High EPA and DHA intake, both of which are n-3 PUFAs, was closely related to a reduction in the level of CRP, which indicated a better prognosis. In addition, a nutritional supplement enriched with n-3 PUFAs has shown advantages in serum ALB levels in patients with head and neck cancer [32]. Vasson [33] confirmed that immunonutrition improves albuminemia in head and neck and esophageal cancer patients undergoing radiochemotherapy. The results of our meta-analysis are in accordance with these reports, in which n-3 PUFAs reduced host inflammatory response by decreasing the concentration of IL-6, TNF-α, and CRP, and improving hypoalbuminemia. The anti-inflammatory response plays an important role in patients with GI cancer [34–36]. N-3 PUFAs may be of benefit in down-regulating the strong and discordant inflammatory response which occurs after surgery.

N-3 PUFAs are beneficial as a dietary supplement in cancer patients as they enhance immune functions. N-3 PUFAs have been recognized as having immuno-modulatory activity, including the activation of T cells and cytokine production [37]. CD4^+^ and CD8^+^ T cells are important effector cells of cell-mediated immunity. CD8^+^ T cells are strong effector T cells. All mature T cells express CD3^+^; CD3^+^ and CD4^+^ T cells are helper T lymphocytes that promote anti-tumor immunity. CD8^+^ cells are suppressor T lymphocytes. Presentation of intracellular antigen on MHC class I molecules activates CD8^+^ T cells, cytotoxic T lymphocytes that will attempt to suppress the intracellular infection. If this does not succeed, the CD8^+^ T cell will kill the target cell by inducing apoptosis or cell lysis. Elevation of CD4^+^/CD8^+^ ratio, CD3^+^ and CD4^+^ lymphocyte percentage were also observed as a result of n-3 PUFAs supplementation. It is essential to understand precisely how specific (n-3) PUFAs modulate immune function. Turbitt [38] suggested that it is possible that n-3 PUFAs induced an increase in IL-2 and IFN-g production in T cells, which may drive a Th1 response, enhance antitumor immunity, and contribute to the cancer prevention effect of n-3 PUFAs. Thus, n-3 PUFA supplementation may enhance Th1 cytokine response and may differentially alter the effector function of T cells. Anita [39] suggested that EPA alone or in combination with 5-FU + Oxaliplatin (FuOx) could be an effective preventive strategy for recurring sporadic colorectal cancer. Cancer stem/stem-like cells (CSCs/CSLCs) are self-renewing undifferentiated cells and are thought to be one of the leading causes of cancer recurrence. EPA acts synergistically with chemotherapy to markedly inhibit the growth of chemo-resistant colon cancer cells which form the bulk of the recurrent tumor. These findings are in accordance with previous evidence that EPA and DHA reduce inflammation in humans and may have anti-neoplastic properties. Kim [40] confirmed that CD4^+^ T-cell proliferation was stimulated by a fish oil diet. The level of CD4^+^ T-cells was higher in the n-3 PUFAs group than in the conventional nutritional support group, indicating that n-3 PUFAs enhanced host immune function. On the other hand, Marano [41] suggested that the intake of n-3 PUFAs improved the immune response by increasing peripheral total lymphocytes, including T lymphocytes, and CD4^+^ T-cells, while several other studies [42–44] suggested negative or inverse results. Different subsets of mature T cells carry out the functions of cell-mediated immunity, including killing virally infected cells and tumor cells (CD8^+^ T cells) and providing help for and regulating components of the immune system (CD4^+^ T cells). Our meta-analysis showed that n-3 PUFAs effectively increased the level of CD3^+^ T cells, CD4^+^ T cells and CD4^+^/CD8^+^ T cells in patients undergoing surgery for GI cancer, but could decrease the level of CD8^+^ T cells, indicating that the immune response was enhanced and rehabilitation was promoted after surgery. Thus modulation of immune responses and reduction of inflammatory responses together lessens postoperative hospital stay for GI cancer patients. And postoperative n-3 PUFAs nutrition for GI cancer is a challenge and need further research.

## Conclusions

Our study has important limitations. The intake of n-3 PUFAs varies considerably within countries, and this may explain the heterogeneity across studies. The outcome estimates were taken from published data; therefore, systematic biases could not be minimized and the data in some cases were incomplete. However, we confirmed that the addition of n-3 fatty acids improved immune function and reduced the level of inflammation in GI cancer patients postoperatively. Thus, despite these limitations and although further larger trials are needed, these fatty acids should be widely used in the clinic.

## Abbreviations

ALB: Albumin
CRP: C-reaction protein
GI: Gastrointestinal
IL-2: Interleukin-2
IL-6: Interleukin-6
n-3 PUFAs: Omega-3 polyunsaturated fatty acids
TNF-α: Tumor necrosis factor-α
